# Antitumor Effects of Curcumin and Glycyrrhetinic Acid-Modified Curcumin-Loaded Cationic Liposome by Intratumoral Administration

**DOI:** 10.1155/2020/4504936

**Published:** 2020-05-30

**Authors:** Mingxiang Chang, Meimei Wu, Hanmin Li

**Affiliations:** ^1^Hubei Key Laboratory of Resources and Chemistry of Chinese Medicine, Hubei University of Chinese Medicine, Wuhan 430061, China; ^2^Hubei Provincial Hospital of Traditional Chinese Medicine, Wuhan 430061, China; ^3^Hubei Province Academy of Traditional Chinese Medicine, Wuhan 430074, China

## Abstract

Curcumin is a hydrophobic polyphenolic compound extracted from the rhizome of *Curcuma longa* and shows a line of active biological functions, but its application has been limited and questioned because of its low solubility, low bioavailability, and rapid metabolism. In terms of antitumor effect, these disadvantages can be overcome by intratumoral injection. In this study, we present the intratumoral injection of curcumin and glycyrrhetinic acid-modified curcumin-loaded cationic liposome (GAMCLCL) in H22 tumor-bearing mice. The experimental results demonstrated that curcumin exhibited positive antitumor activities in vitro and in vivo by intratumoral injection, but its activities were much weaker than GAMCLCL and adriamycin. Compared with free curcumin, GAMCLCL showed much better effects in improving the blood parameters (WBC, RBC, PLT, ALT, CRE, and LDH), inhibiting tumor growth, reducing tumor microvascular density, downregulating the expression of VEGF-protein and mRNA, and upregulating the expression of caspase-3 protein and mRNA in H22 tumor tissues. Under the experimental conditions of this study, the antitumor effect of high-dose GAMCLCL was similar to adriamycin. In conclusion, the experimental results demonstrated that free curcumin possessed definite antitumor efficacy, but its antitumor activities were weaker, and some strategies should be adopted to overcome its disadvantages, improve, and ensure its clinical efficacy.

## 1. Introduction

Curcumin is a natural polyphenolic compound extracted from the rhizome of *Curcuma longa*, which has been used as traditional Chinese medicine in China and as curry in India for over centuries. Curcumin is “generally recognized as safe” by the Food and Drug Administration (FDA). A line of studies indicate that curcumin has shown good activities for cancers, such as liver cancer, breast cancer, gastric cancer, colorectal cancer, esophageal cancer, skin cancer, lymphoma, and leukemia, in experimental animal systems [1–3]. The USA National Cancer Institute (NCI) had listed it as a third generation of cancer chemopreventive drugs [4].

However, curcumin exists some disadvantages: ① poor water solubility, chemical instability, photoinstability, poor bioavailability, and rapid metabolism [5]; ② weak antitumor activity (compared with usual chemotherapy drugs); ③ unclear clinical efficacy. These disadvantages limit its utilization as an effective therapeutic agent, and especially, its activities were recently questioned and considered to be deceptive [6, 7].

In effort to address the above limitations ① and ② of curcumin, in a previous study, we prepared curcumin into the glycyrrhetinic acid-modified curcumin-loaded cationic liposome (GAMCLCL) and mainly presented their antitumor efficacy by tail vein injection [8].

However, in treatment of cancer, many lines of evidences show that drugs are often limited to the periphery of the tumor mass [9, 10]. The reason is that many solid tumors develop several biological features distinguished from those of normal tissues [11,12], such as increased stiffness of tumor extracellular matrix (ECM) and relatively high interstitial fluid pressure (IFP) [13, 14]. The high IFP and abnormal ECM structure are known to be significant barriers to drug diffusion into the tumor mass, so the core part of the tumor usually remains unaffected and becomes a potential source for tumor relapse or metastasis. In order to overcome the barriers of tumor mass and to reduce adverse effects, the intratumoral chemotherapy has been promoted by many groups [15, 16], and in some cases, locoregional delivery of liposomal agents by intratumoral injection has been proposed as a strategy to eliminate the anatomic and physiological barriers [17].

In addition, intratumoral injection is also frequently performed in the preclinical steps on animal models to develop and assess the efficacy of new therapies [18, 19].

In particular, in terms of antitumor effect, intratumoral injection can achieve the effect of allowing the drug to enter directly inside the solid tumor, without drug absorption process, and can overcome the problems of poor bioavailability, rapid metabolism, and poor pharmacokinetics of drugs. Thus, in this paper, we mainly explored the antitumor activities and potential mechanism of curcumin and GAMCLC by intratumoral injection.

## 2. Materials and Methods

### 2.1. Reagents

Curcumin was purchased from Hangzhou Sky Grass Technology Company Ltd. (Hangzhou, China). GA was from Hubei Yuanda Pharmaceutical Industry (Wuhan, China). Octadecylamine was from Sigma Company (USA). The complex of GA and octadecylamine (CGO) was synthesized in our laboratory. Adriamycin was from Shenzhen Main Luck Pharmaceuticals Inc. (Shenzhen, China). Lecithin was from Shanghai AVT Technology Pharmaceutical Ltd. (Shanghai, China). Pancreatic enzymes were obtained from Gibco Company (USA).

### 2.2. Cell and Animals

The H22 cell line (CL-0341) was obtained from Procell Company (Wuhan, China).

Male Kunming mice (weight 20 ± 2.0 g) were obtained from the Medical Animal Test Center of the Hubei Disease Control Center (Wuhan, China).

### 2.3. Preparation of GAMCLCL

The GAMCLCL was prepared by referring a previous report [8]. On a 50°C water bath, CGO (25 mg), lecithin (200 mg), and curcumin (8 mg) were dissolved in 3 mL anhydrous ethanol and kept warm for 10 min. 1 mL of the residue solution was injected slowly and uniformly into 20 ml of preheated and stirred double-distilled water. The solution was stirred (250 rpm) at 50°C until the ethanol was completely evaporated, then kept at room temperature for 24 h, filtered through a 200 nm microporous membrane to obtain GAMCLCL, and then stored at 4°C in a sealed container.

The morphological characteristics and the entrapment efficiency of GAMCLCL were determined referring a previous report [8].

### 2.4. Cytotoxicity Assay

Curcumin and GAMCLCL were added to H22 cells at various concentrations and incubated for 24 and 48 h, respectively. The cytotoxicity was evaluated by using the CCK-8 assay kit (Biosharp, Hefei, China) following the manufacturer's instructions.

### 2.5. Apoptosis Analysis of H22 Cells

The H22 cells were treated with free curcumin (10 *μ*g/mL), GAMCLCL (equivalent to containing 10 *μ*g/mL curcumin), and blank (only 1640 medium). The Annexin V-APC/7-AAD (KGA1026, Nanjing, China) kit was used to check the apoptosis following the manufacturer's instructions.

### 2.6. Antitumor Effect of Curcumin

Under sterile conditions, the mice were vaccinated using the H22 cell line in the right fore axillary region of each mouse and then fed under normal conditions. After 5 days of vaccination, the mice were randomly divided into three groups (*n* = 6) and administrated by intratumoral injection of curcumin every day for 7 days: ① the model group was injected with saline at the same volume as the other groups; ② the curcumin groupI was injected with 20 mg/kg curcumin; ③ the curcumin groupII was injected with 40 mg/kg curcumin (curcumin was dissolved in DMSO and diluted with water to the desired concentration; the concentration of DMSO <1%).

On the eighth day, all mice were weighed and sacrificed by cervical dislocation and then the tumor of each mouse was separated and weighed.

The tumor growth inhibition rate (IR) was calculated by the following formula: IR = [(*A* − *B*)/*A*] × 100% (*A* is the average tumor weight of the model group; *B* is the tumor weight of the treated group).

### 2.7. Antitumor Effect of GAMCLCL

Model preparation was the same as in Section 2.6, treated as follows: ① the model group was injected with saline at the same volume as the other groups; ② the adriamycin group was injected with 1 mg/kg adriamycin; ③ the curcumin group was injected with 20 mg/kg curcumin (diluted in DMSO); ④ the high-, ⑤ the middle-, and ⑥ the low-dose GAMCLCL groups were injected with 20, 10, and 5 mg/kg (containing curcumin) of GAMCLCL solution, respectively. During the experimental period, the routine actions of the mice were observed and recorded.

On the eighth day, all mice were weighed and sacrificed by cervical dislocation and then the tumor of each mouse was separated and weighed. The blood samples and tumor samples of these experimental mice were collected and checked as follows.

### 2.8. Blood Biochemical Examination

Continuing under Section 2.7, the blood samples of mice were collected on the eighth day, and the serum samples were harvested by using centrifugation. The red blood cell (RBC) count, white blood cell (WBC) count, and platelet (PLT) count were measured in the blood samples, and alanine aminotransferase (ALT) and creatinine (CRE) were evaluated in the serum samples by using an automatic biochemical analyzer (Hitachi 7020, Japan). Lactate dehydrogenase (LDH) in the serum samples was determined by using LDH kit.

### 2.9. H&E Staining of Tumor Mass

The tumor tissue was fixed for 48 hours in 4% paraformaldehyde solution, dehydrated, and embedded in rosin. Sections were cut to a thickness of 5 *μ*m, stained with hematoxylin and eosin (H&E), and then subjected to a histopathological examination by using a microscope.

### 2.10. Apoptosis of Tumor Tissue

After dehydration and transparency, tumor tissues were embedded in wax, the paraffin slices attached to the slide were dewaxed in a 60°C oven, and then the tunel method was used to check the apoptosis following the instructions. Five high-power fields (400 times) were selected, and the number of all cells and the number of apoptotic cells were counted in each field.

The tunel labeling index (LI) = the number of positive cells in each field of view/the number of all cells in the field of view.

The apoptotic index (AI) of each tumor tissue = the average of five labeling indexes.

### 2.11. Tumor Microvascular Density Assay

Tumor microvascular density (MVD) was checked by using the Immuno-Bridge + kit (GBI Company, USA) following the manufacturer's instructions.

### 2.12. Western Blot of VEGF and Caspase-3

A small piece of tumor tissue from each mouse was collected, grounded, cracked, and centrifuged. The protein concentrations of each diluted sample were determined by using BCA assay kit. The total protein (40 *μ*g) and MAKER were resolved by electrophoresis and transferred to PVDF membranes. The conditions for membrane transfer were as follows: VEGF, 200 mA and 70 min; caspase-3 and beta actin, 200 mA and 90 min. The membranes were blocked in TBST containing 5% skimmed milk for 2 hours at room temperature and then incubated with anti-caspase-3 antibody (1 : 800 dilution in TBST, Proteintech Group Inc., China) and anti-VEGF antibody (1 : 600 dilution in TBST, Bioworld, USA) overnight at 4°C. After five full washes in TBST, the membranes were incubated with anti-mouse IgG antibody labeled with horseradish peroxidase (1 : 50000 dilution in TBST, Boster, China) for 2 hours. The membrane was exposed to the X-ray film, which was rinsed, dried, and scanned, and then the grey value of the film was computed by using BandScan software.

### 2.13. RT-PCR

The Trizol method was used to extract total RNA from the tumor tissue in each group. The RNA concentration and purity were detected by using a microspectrophotometer (Hangzhou Allsheng Instruments Co., Ltd., Hangzhou, China). In accordance with the VAZVME kit instruction, the first-strand cDNA was synthesized by Oligo-dT reverse transcription. The sequences of the VEGF and caspase-3 coding regions were searched in the NCBI, the primers were designed by using primer3.0, and the best primers were screened by using Blast software.

 
*β*-Actin: forward primer: CACGATGGAGGGGCCGGACTCATC and
  reverse primer: TAAAGACCTCTATGCCAACACAG-T.
  VEGF: forward primer: CATCTTCAAGCCGTCCTGTG and
  reverse primer: GACCCTTTCCCTTTCCTCGA.
  Caspase-3: forward primer: CAGCCAACCTCAGAGAGACA and
  reverse primer: ACAGGCCCATTTGTCCCATA.

The QRT-PCR experiment was performed by using a ViiA7 real-time fluorescence quantitative PCR instrument (ABI7900/Illumina Eco, Applied Biosystems Company, USA), and data were subsequently exported for analysis.

### 2.14. Statistical Analysis

All quantitative data are generated as mean ± standard deviation (S.D.). The *t*-test was used to test the differences between groups. The significance level was set at a value of *P* < 0.05. *P* < 0.01 indicated the extremely significant difference.

## 3. Results

### 3.1. The Characteristics of GAMCLCL

**Figure 1:**
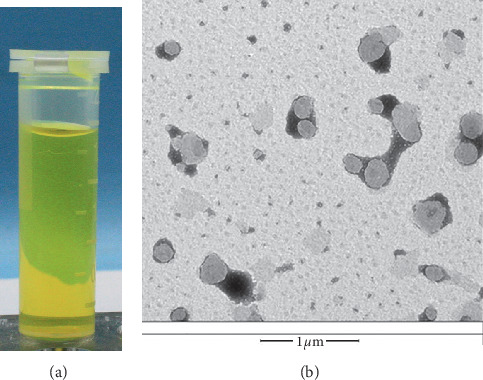
(a) The solution of GAMCLCL; (b) the morphology of GAMCLCL (magnifying 15000 times).

GAMCLCL formed a clear, yellow, colloidal, and stable solution (Figure 1), and this indicated that GAMCLCL improved the solubility of curcumin, for curcumin is hardly soluble in water and precipitates in water. The particle size was 194 ± 0.25 nm, the potential was 31.9 ± 0.31 mv, and the entrapment efficiency was 98.26 ± 1.33%.

### 3.2. Cytotoxicity In Vitro

**Figure 2:**
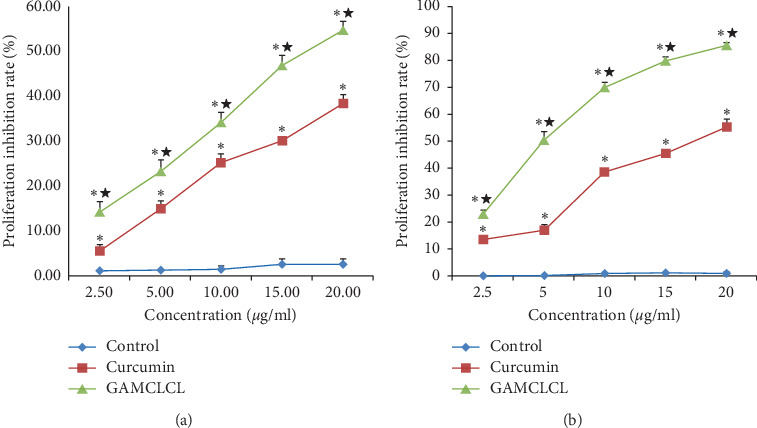
The cytotoxicity of different drugs on H22 cells treated for (a) 24 h and (b) 48 h, determined by CCK-8 assay kit. Data are shown as means ± SD. Compared with the control group, ^*∗*^*P* < 0.05 and ^**★**^*P* < 0.01.

As shown in Figure 2, compared to the curcumin-treated group, the cell proliferation inhibition rate in the GAMCLCL-treated group was dramatically increased at 24 and 48 h. However, the blank liposomes (the control) only exerted a slight inhibitory effect on H22 cell proliferation, which indicated that the blank liposomes induced hardly any cytotoxic effects.

### 3.3. Cellular Apoptosis Results In Vitro

**Figure 3:**
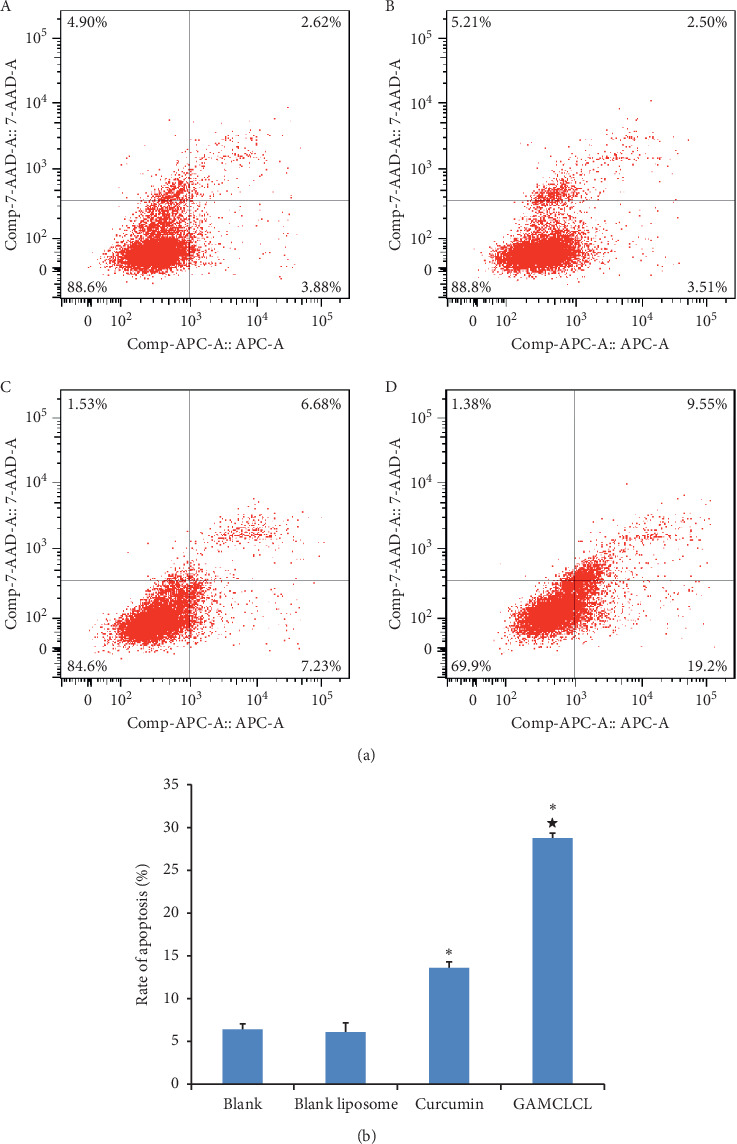
The results of cellular apoptosis. (a) Photograph of cellular apoptosis of different drugs by flow cytometry. (b) The bar graph of the apoptosis rate of different drugs. Data are shown as means ± SD. Compared with the control group, ^*∗*^*P* < 0.05; compared with the curcumin group, ^**★**^*P* < 0.01.

The results of cellular apoptosis are shown in Figure 3. Compared to the blank group, the apoptosis of H22 cells was significantly increased by treatment with GAMCLCL and free curcumin (*P* < 0.01), and the cellular apoptosis induced by GAMCLCL was much stronger than that of free curcumin (*P* < 0.01).

### 3.4. Antitumor Efficacy

**Figure 4:**
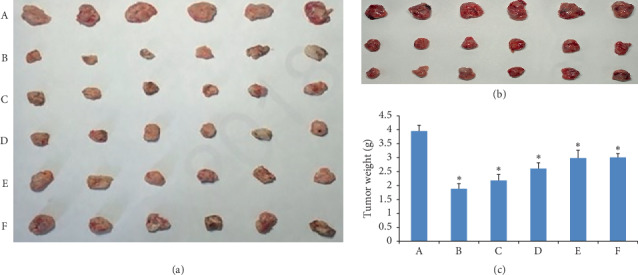
(a) Tumor morphology of intratumoral injection of GAMCLCL; (b) tumor morphology of intratumoral injection of curcumin (the model group, 20 mg/kg group, and 40 mg/kg group); (c) the bar graph of tumor weight by intratumoral injection of GAMCLCL. (A) Model group; (B) adriamycin group; (C) high-dose GAMCLCL group; (D) middle-dose GAMCLCL group; (E) low-dose GAMCLCL group; (F) curcumin group. Data are shown as means ± SD. Compared with the control group, ^*∗*^*P* < 0.01.

The tumor morphology of intratumoral injection of curcumin is shown in Figure 4(b) (three groups, the model group, 20 mg/kg group, and 40 mg/kg group). The tumor inhibition rates of the injection of 20 mg/kg and 40 mg/kg were 38.5% and 43.1%, respectively. Since the tumor inhibition rate of the two doses was not much different, we chose the dose of 20 mg/kg for the following test.

The tumor morphology in each treatment group by injection of GAMCLCL is shown in Figure 4(a), which provided an intuitive antitumor efficacy of different agents: the smaller the tumor size was, the stronger the antitumor effect was. The tumor size of adriamycin- and high-dose GAMCLCL-treated groups was obviously smaller than that of the other treatment groups.

The tumor weights of GAMCLCL-treated groups are shown in Figure 4(c). Compared to the model group, the tumor weight of mice in each treatment group was significantly reduced (*P* < 0.01). The tumor weight of three groups treated with GAMCLCL was negatively correlated with the dose. The tumor weight of the mice treated with high-dose GAMCLCL had no statistical difference with the adriamycin-treated mice (*P* > 0.05), which showed that the antitumor effect of high-dose GAMCLCL was similar to adriamycin.

Nevertheless, the tumor weight treated with curcumin was much larger than the adriamycin-treated group, which indicated that the antitumor effect of curcumin was much weaker than that of adriamycin.

All mice survived well and had normal life after intratumoral injection of GAMCLCL for 7 days, indicating that GAMCLCL can be injected intratumorally.

### 3.5. Blood Biochemical Results

**Figure 5:**
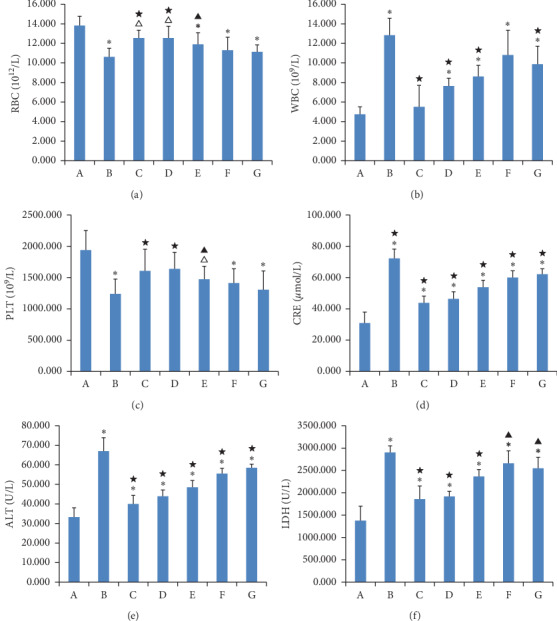
Bar graph of WBC, RBC, PLT, CRE, ALT, and LDH. (a) Normal group; (b) model group; (c) adriamycin group; (d) high-dose GAMCLCL group; (e) middle-dose GAMCLCL group; (f) low-dose GAMCLCL group; (g) curcumin group. Data are shown as means ± SD. Compared with the normal group, ^Δ^*P* < 0.05 and ^*∗*^*P* < 0.01; compared with the model group, ^**▲**^*P* < 0.01 and ^**★**^*P* < 0.01.

The results of blood biochemical tests are shown in Figure 5. Compared to the normal group, the RBC counts in the model group, the curcumin-treated group, and the three GAMCLCL-treated groups were significantly decreased (*P* < 0.05 or *P* < 0.01), but the reduction observed in the adriamycin-treated group (the positive control group) was not statistically significant (*P* > 0.05). The RBC counts of the three GAMCLCL-treated groups were positively correlated with the treatment dose. Compared to the model group, the reduction in adriamycin-treated and the high-dose GAMCLCL-treated groups represented a significant amelioration of the decreased RBC count (*P* < 0.05 and *P* < 0.01, respectively). The RBC count of the high-dose GAMCLCL-treated group was similar to the adriamycin-treated group, with no statistical difference found between the two groups (*P* > 0.05).

In contrast, the WBC count was significantly increased in the model group, the curcumin group, and the three GAMCLCL-treated groups in comparison with the normal group (*P* < 0.01). The WBC count was highest in the model group, and no statistically significant difference was observed between the adriamycin group and the normal group. The changes in the WBC count of the three GAMCLCL-treated groups were negatively correlated with the GAMCLCL dose. Compared to the model group, all treatment groups cut down an increased WBC count, to varying degrees (*P* < 0.05 or *P* < 0.01).

The changes in the PLT count of all groups were similar to those of RBC.

ALT, CRE, and LDH are related to the function of the liver, kidney, and heart, respectively. Compared to the normal group, the ALT, CRE, and LDH of all H22 tumor-bearing mouse groups significantly increased (*P* < 0.01) and the ALT, CRE, and LDH values in H22 tumor-bearing mice were significantly increased. The values were highest in the model group, which indicated that the functions of the heart, liver and kidney in H22 tumor-bearing mice were damaged. Nevertheless, the ALT, CRE, and LDH values in each treatment group were significantly reduced compared to the model group (*P* < 0.05 or *P* < 0.01). The ALT, CRE, and LDH values of the three GAMCLCL-treated groups were negatively correlated with the dose; furthermore, the reduction of CRE and LDH in the high-dose GAMCLCL group was not significantly different from the adriamycin group, which indicated that GAMCLCL clearly improved the functions of the heart, liver, and kidney of H22 tumor-bearing mice.

### 3.6. Histological Changes of Tumor Tissue

**Figure 6:**
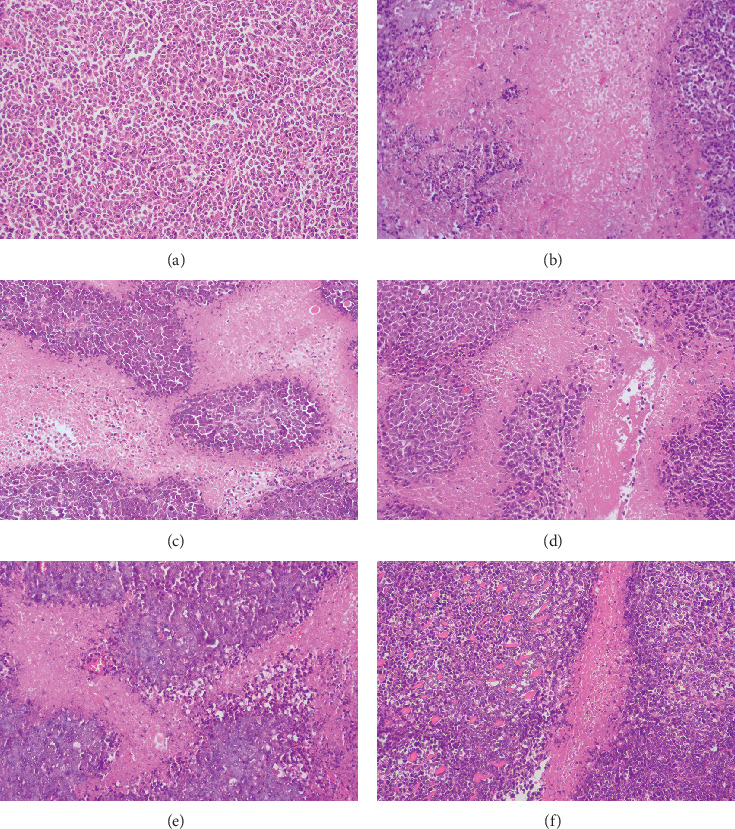
Microscope pictures of tumor slices of different groups by H&E staining (magnifying 200 times). (a) Model group; (b) adriamycin group; (c) high-dose GAMCLCL group; (d) middle-dose GAMCLCL group; (e) low-dose GAMCLCL group; (f) curcumin group.

The tumor pathology of H&E stained sections was examined by microscopy (Figure 6). The tumor cells in the model group were closely packed, displayed diffuse growth, and showed an irregular cell shape with large nuclei, no obvious signs of necrosis, and a small number of interstitial cells spread between the tumor cells. In the drug administration groups, the tumor cell gap increased, with evidence of nucleus pyknosis, a clear area of necrosis, and sparse cell distribution. Treatment with adriamycin and high- and middle-dose GAMCLCLs resulted in a larger necrosis area of the tumor cells, and treatment with low-dose GAMCLCL and curcumin resulted in a smaller necrosis area. The histopathological analysis of the tumor slices demonstrated that the curcumin and GAMCLCL directly destroyed the tumor tissue.

### 3.7. The Apoptotic Index of Tumor Tissue

**Figure 7:**
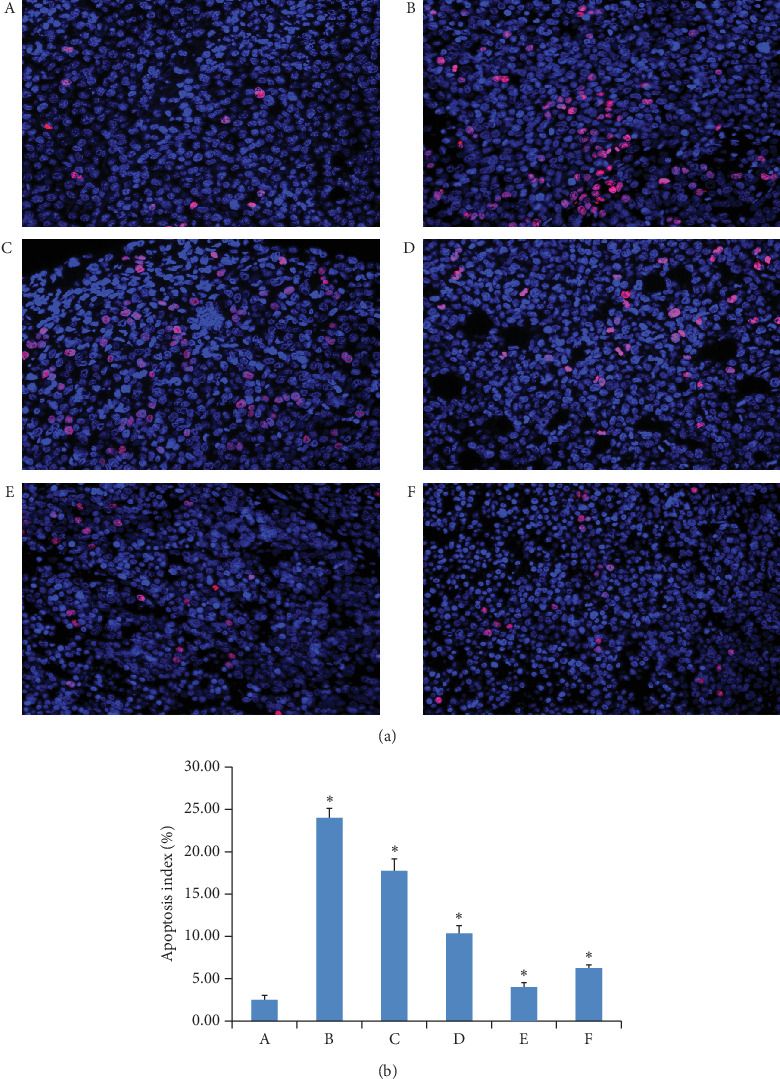
(a) The apoptosis pictures of tumor tissue of different groups (magnifying 400 times). (b) The bar graph of apoptotic index of tumor tissue in each group. (A) Model group; (B) adriamycin group; (C) high-dose GAMCLCL group; (D) middle-dose GAMCLCL group; (E) low-dose GAMCLCL group; (F) curcumin group. Data are shown as means ± SD. Compared with the model group, ^*∗*^*P* < 0.01.

The apoptosis index (AI) of tumor tissues in each group is shown in Figure 7. The apoptosis index (AI) reflects the effect of drug-inducing apoptosis of tumor tissue. Compared with the model group, the AI of each administration group was significantly increased, and AI of GAMCLCL-treated groups increased and positively correlated with the dose of GAMCLCL.

### 3.8. Results of MVD

**Figure 8:**
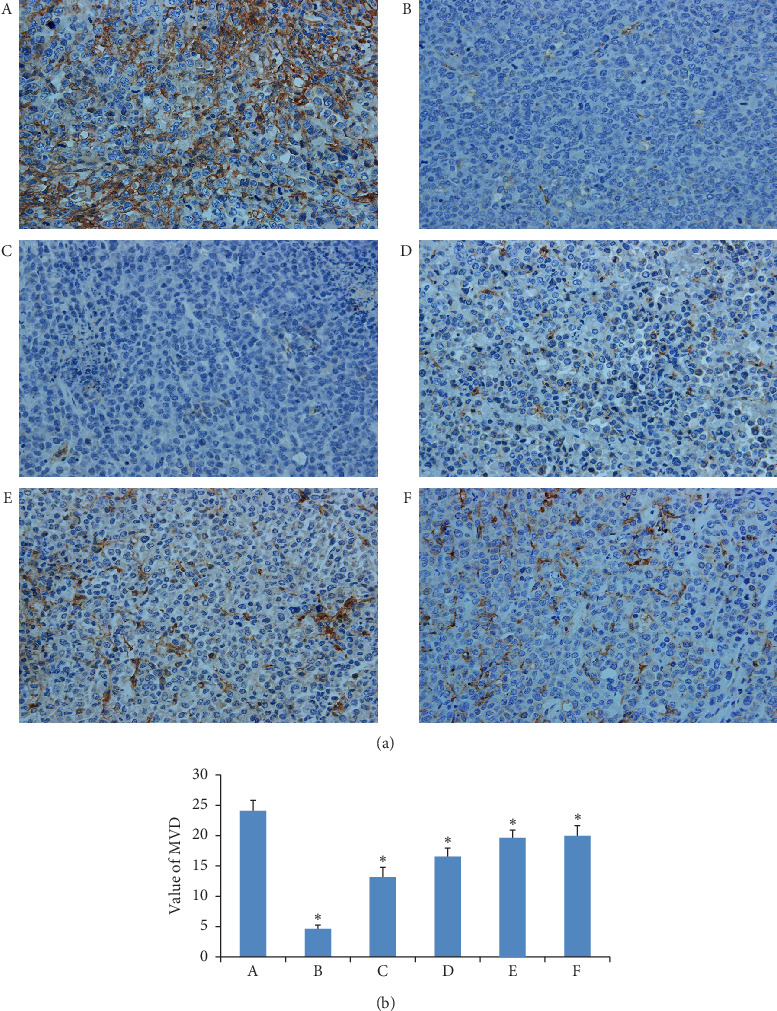
(a) The immunohistochemical pictures of MVD in tumor tissues of experimental groups (magnifying 400 times). (b) The bar graph of MVD of tumor tissues in experimental groups. (A) Model group, (B) adriamycin group, (C) high-dose GAMCLCL group, (D) middle-dose GAMCLCL group, (E) low-dose GAMCLCL group, and (F) curcumin group. Data are shown as means ± SD. Compared with the model group, ^*∗*^*P* < 0.01.

The immunohistochemical pictures of MVD are shown in Figure 8, and the brown regions indicate angiogenesis. The brown regions in the model group were markedly greater than those in other groups. The MVD values are presented in a bar graph in Figure 8. Compared to the model group, the MVD of the curcumin group was not obviously different, but the values in the other drug administration groups were significantly decreased (*P* < 0.05 or *P* < 0.01). The MVD values of the three GANCLCL groups were negatively correlated with the dose.

### 3.9. VEGF and Caspase-3 Protein Expression

**Figure 9:**
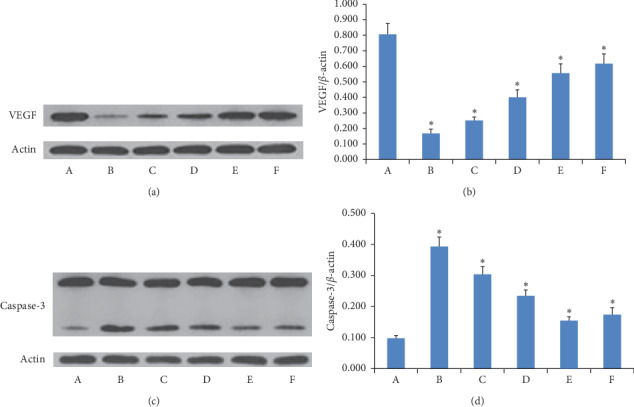
(a) and (c) The electropherogram of VEGF and caspase-3 in experimental groups. (b) and (d) The bar graph of VEGF and caspase-3 protein expression in experimental groups. (A) Model group; (B) adriamycin group; (C) high-dose GAMCLCL group; (D) middle-dose GAMCLCL group; (E) low-dose GAMCLCL group; (F) curcumin group. Data are shown as means ± SD. Compared with the model group, ^*∗*^*P* < 0.01.

The western blot for the VEGF and caspase-3 protein is shown in Figure 9, and the intensity of the bands for VEGF and caspase-3 indicated their expression levels. Compared to the model group, the expressions of VEGF were significantly decreased in each treatment group (*P* < 0.05 or *P* < 0.01). The effect of the high-dose GAMCLCL group was similar to the effect of the adriamycin group. VEGF expression in the three GANCLCL groups was negatively correlated with the drug dose.

In contrast, compared to the model group, the expressions of caspase-3 were significantly increased in each treatment group (*P* < 0.05 or *P* < 0.01). The caspase-3 expression in the three GANCLCL groups was increased as the dose increased.

### 3.10. VEGF and Caspase-3 mRNA Expression

**Figure 10:**
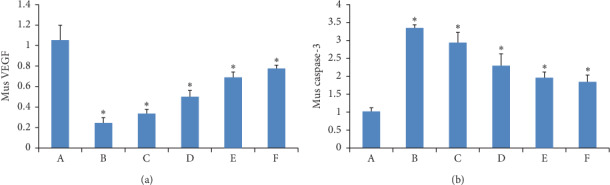
The bar graph of the mRNA expression of (a) VEGF and (b) caspase-3 in H22 tumor-bearing mice. Compared to the model group, ^*∗*^*P* < 0.01.

The mRNA expressions of VEGF and caspase-3 in H22-bearing mice were determined by quantitative RT-PCR. As shown in Figure 10, compared to the model group, the mRNA expression of VEGF in each treatment group was significantly decreased (*P* < 0.05 or *P* < 0.01), and the mRNA expression of VEGF in the three GANCLCL groups decreased as the dose increased.

Compared to the model group, the mRNA expressions of caspase-3 in each treatment group were significantly increased (*P* < 0.05 or *P* < 0.01), and the caspase-3 mRNA expression in the three GANCLCL groups was positively correlated with the drug dose.

A comparison of the bar graphs of Figures 9 and 10 revealed that the changes in VEGF-protein expression in each group were very similar to those of VEGF-mRNA expression in each group, this pattern was also observed for caspase-3.

## 4. Discussion

In recent years, cancer incidence and mortality are rapidly growing worldwide. Cancer is expected to be the leading cause of death and the most important barrier to increasing life expectancy in the 21st century. According to the statistics of the World Health Organization (WHO) in 2015, cancer is the first or second leading cause of death before age 70 years in 91 of 172 countries and the third or fourth in an additional 22 countries [20]. Therefore, treatment for cancer is still an important issue in international research.

At present, chemotherapy is still one of the important methods for the treatment of cancer. However, the majority of chemotherapeutic drugs are cytotoxic molecules, upon systemic administration, and these drugs are generally distributed within the whole body and may result in toxicity to normal tissues [21]. So, drug-targeting delivery has become an important method in experimental and clinical research. As one of the cancer targeted drug delivery methods, intratumoral intervention treatment can increase antitumor efficacy and reduce side effects, which has played an important role in the treatment of tumor.

In fact, intratumoral administration of drugs has been a treatment option for many years [22]. In addition, intratumoral injection is suitable for patients who cannot be treated with whole body chemotherapy and local surgery for some other advantages of a minimally invasive route, less complications, and low operational risk [23].

A great deal of studies show that intratumoral injection of drugs has been proven to have a positive effect after many years of clinical research, can provide high drug concentrations at the tumor site with minimal exposure of nontarget tissues, and can prolong patient survival and improve quality of life [24–26]. Since Sugiura [27] first reported that PEI (percutaneous alcohol injection therapy) was adopted to treat liver cancer in 1983, the related research and clinical applications in the field have attracted wide attention. For primary or recurrent small liver cancer with a tumor number ≤3 and a tumor size ≤3 cm, the effect of PEI was similar to that of surgical operation [28].

Moreover, the cationic liposome carries positive charge, which can achieve electrostatic binding with the surface of the negatively charged tumor cells, increase the local retention of the preparation in the tumor tissue, and reduce the distribution to other tissues and the side effects. Nomura showed that positively charged liposomes increased the retention of the preparation in tumor tissues [29]. Ueno NT reported that intratumoral administration gained favor to deliver cationic liposome-based gene therapies to shift the organ distribution and increase tumor liposome concentrations [30].

The GAMCLCL carried positive charge, and intratumoral injection of GAMCLCL should possess some advantages; thus, in this study, we mainly presented the antitumor efficacy of free curcumin and GAMCLCL by intratumoral injection.

In vitro, the experimental results demonstrated that curcumin promoted apoptosis of H22 cells and enhanced the inhibition of H22 cell proliferation. Compared to free curcumin, GAMCLCL exhibited a stronger inhibitory effect on H22 cell. These results showed that curcumin and GAMCLCL directly inhibited the H22 cells in vitro.

In vivo, curcumin showed definite antitumor activities by intratumoral injection. The tumor inhibition rates of the injection of 20 mg/kg and 40 mg/kg were 38.5% and 43.1%, respectively.

The histopathological analysis of the tumor slices demonstrated that the curcumin and GAMCLCL could directly destroy the tumor tissue, and GAMCLCL exhibited stronger effects.

In vivo, the antitumor activities of GAMCLCL followed in a dose-dependent manner. Compared with free curcumin, GAMCLCL exhibited much better effects in improving the parameters of WBC, RBC, ALT, CRE, and LDH, inhibiting tumor growth, inducing apoptosis of tumor tissue, reducing MVD, downregulating the expression of VEGF-protein and mRNA, and upregulating the expression of caspase-3 protein and mRNA in H22 tumor tissues. The antitumor activities of curcumin were much weaker than those of adriamycin, but under the experimental conditions of this study, the antitumor effect of high-dose GAMCLCL was similar to adriamycin (*P* > 0.05).

It is widely accepted that angiogenesis is essential for tumor growth and invasion, and inhibition of angiogenesis is one of the important methods for treatment of cancer [25]. MVD reflects the tumor microvascular formation and the tumor aggression. Numerous studies have confirmed that the MVD is associated with the tumor growth rate and the survival term of patients [31]. VEGF is the strongest vasoactive factor and induces the formation of new capillaries from nearby normal existing ones [32]. Caspase-3 is the main executor of the apoptosis, the activation of caspase-3 is an important mechanism of cell apoptosis, and the inactivation or anomalies expression is related to the occurrence and development of a wide variety of tumors [33]. Thus, curcumin and GAMCLCL could not only directly affect the H22 cells and tumor tissue but also play an antitumor effect by inhibiting tumor angiogenesis and inducing tumor cell apoptosis.

Although curcumin showed a line of active biological functions, such as antitumor, anti-inflammatory, antioxidant, antimicrobial, anti-Alzheimer, antidiabetic, and antirheumatic activities [34], its activities were recently considered to be deceptive [6]. Baker reported that there is no evidence that it had any specific therapeutic benefits, despite thousands of research papers and more than 120 clinical trials [7].

We think that the reasons being questioned may be as follows: ① its weak antitumor efficacy. In this paper, the results showed that its antitumor efficacy was much weaker than adriamycin (the first-line chemotherapy drug). ② its poor water solubility, instability, poor bioavailability, and rapid metabolism. A pharmacokinetics study in 12 healthy human volunteers showed that after an oral dose of 10 or 12 g, with the assay limit of a detection of 50 ng/mL, only 1 subject had detected the free curcumin at any of the time points [35]. A clinical study comprised of 15 colorectal cancer patients showed that the cancer was nonresponsive to curcumin at a daily dose of 3.6 g for 4 months [36]. In addition, curcumin was rapidly metabolized into tetrahydrocurcumin and hexahydrocurcumin, for extensive metabolism in the intestine and liver, and high concentrations of curcumin could not be achieved and maintained in plasma and tissues after oral administration [37]. Above studies could maybe explain the reasons of its effect being questioned.

In terms of cancer, intratumoral injection can deliver drugs directly inside the solid tumor, without absorption process, and overcome the problems of poor bioavailability, rapid metabolism, and poor pharmacokinetics of curcumin; thus, the results obtained by intratumoral injection should truly reveal the antitumor effect of curcumin. In this paper, the tumor inhibition rates of intratumoral injection of 20 mg/kg and 40 mg/kg were 38.5% and 43.1%, respectively, which could demonstrate the antitumor effect of curcumin.

When intratumoral injection of curcumin with 20 mg/kg and 40 mg/kg, for the average weight of a mouse is about 20 g, the true injection dose per mouse of the both groups was approximately 400 *μ*g and 800 *μ*g, respectively. In this study, the volume per tumor mass of both groups was approximately 2900 mm^3^ and 2700 mm^3^, so the corresponding concentrations of curcumin in tumor mass were approximately 138 *μ*g/mL and 296 *μ*g/mL (1 mL = 1000 mm^3^, assuming evenly distributed within the tumor), respectively. Comparing with [35], in fact, the curcumin concentrations of 138 *μ*g/mL and 296 *μ*g/mL in tumor mass were difficult to achieve by other administration methods, which could also explain why its therapeutic benefits were questioned by oral administration.

In conclusion, the experimental results demonstrated that free curcumin possessed definite antitumor efficacy, but its antitumor activities were weaker, and some strategies should be adopted to overcome its disadvantages, improve, and ensure its clinical efficacy.

## 5. Conclusions

In this paper, the experimental results confirmed the antitumor effect of curcumin and GAMCLCL in vitro and in vivo by intratumoral injection, but the antitumor activities of curcumin were much weaker than adriamycin and GAMCLCL. The concentrations of curcumin in tumor mass by intratumoral injection were difficult to achieve by other administration methods. The experimental results also revealed that the antitumor effect of curcumin could be significantly enhanced by making curcumin into GAMCLCL and by intratumoral injection.

## Data Availability

All data generated or analyzed during this study are included within this article. However, further details are available from the corresponding author on reasonable request.

## Abbreviations

GAMCLCL: Glycyrrhetinic acid-modified curcumin-loaded cationic liposome
IR: The tumor growth inhibition rate
RBC: Red blood cell
WBC: White blood cell
PLT: Platelet
CRE: Creatinine
LDH: Lactate dehydrogenase
ALT: Alanine aminotransferase
MVD: Tumor microvascular density
AI: Apoptotic index
LI: The tunel labeling index
VEGF: Vascular endothelial growth factor
PEI: Percutaneous alcohol injection therapy.
